# Climatic factors driving *influenza* transmission in Sahelian area: A twelve-year retrospective study in Niger (2010–2021)

**DOI:** 10.1371/journal.pone.0322288

**Published:** 2025-05-08

**Authors:** Adamou Lagare, Emeline Perthame, Ramatoulaye H. Lazoumar, Fakany A. Aboutalib, Bintou Kiari Kaka, Bibata Abdou Sidikou, Bassira Issaka, Katoumi Moumouni, Jean Testa, Ronan Jambou

**Affiliations:** 1 Centre de Recherche Médicale et Sanitaire (CERMES), Niamey, Niger; 2 Institut Pasteur, Université Paris Cité, Bioinformatics and Biostatistics Hub, Paris, France; 3 Ministère de la santé publique, Niamey, Niger; 4 Université Nazi Boni, Bobo-Dioulasso, Burkina Faso; 5 Institut Pasteur Global Health department, Paris, France; Satyawati College, University of Delhi, INDIA

## Abstract

The relationship between influenza transmission and climate has many public health implications, particularly on the occurrence of epidemics and disease severity. Environmental factors such as temperature, wind and humidity can influence transmission, particularly in this time of climate change. This study aims to use statistical modelling to decipher the impact of climate factors on influenza transmission in Niger. The reference center of respiratory disease (CERMES) collected samples from patients with acute respiratory illness in eight sentinel sites over a period of twelve years. Detection of respiratory virus was conducted on each sample using molecular approaches. Meteorological parameters were recorded on a weekly basis at the National Meteorological Station in Niamey. Climatic and virological data were plotted over the weeks of the years. A multivariate approach was used to identify clusters of weeks with homogeneous climatic conditions, independent of the season. The impact of the predictor variables was determined using generalized additive modelling (GAM). During this study, 9836 suspected influenza cases were PCR tested, of which 982 (9.98%) were confirmed positive for either influenza A or B. 631 (64.25%) of the influenza A/B positive cases were detected during the low temperature periods (December to February). Using clustering analysis, six distinct periods can be identified, with the most favorable conditions for influenza occurring in conjunction with dry, cold and windy weather patterns. Of greater importance, however, are the conditions that predominate in the weeks preceding the detection of clinical cases. The final GAM model accounts for 77% of the variability in the occurrence of influenza cases, indicating that the epidemic can be anticipated weeks before clinical detection in dispensaries using wind and minimum temperature as indicators. Clustering and GAM models can be considered as an efficient and simple approach to analyze the impact of climatic conditions on the transmission of infectious diseases.

## Introduction

Acute respiratory infections (ARI) represent a significant global health and economic burden on healthcare systems, with high rates of morbidity and mortality [1,2]. It is estimated that between 11 and 22% of deaths among children under five years and 3% of deaths among adults aged 15–49 years globally are attributable to acute respiratory infections (ARI) [3]. Viral pathogens represent the primary etiology of ARI, including the influenza virus, respiratory syncytial virus (RSV), rhinovirus (RV), and most recently, SARS-CoV-2 [4]. While there are similarities in the clinical syndromes caused by viruses capable of infecting and causing disease in the human respiratory tract, the transmission patterns among humans vary considerably [5]. The interaction between the host, pathogen, and environment may be influenced by climatic factors, which could potentially increase the probability of exposure, susceptibility, and infection [6]. It is hypothesized that environmental factors, including temperature, wind direction, humidity, pollution, and ultraviolet radiation, influence the transmission of these respiratory viruses [7–9]. Furthermore, extreme weather conditions resulting from climate changes may also modulate respiratory virus transmission. These viruses are most prevalent during the winter in temperate zones [10,11], and for example in Europe, RSV infections have been linked to cooler temperatures and greater humidity [12]. In tropical regions, such conditions can be observed year-round and are not only limited to a specific season. A preliminary study conducted in Niger Republic, a Sahelian country characterized by high temperatures and low humidity, as part of the national influenza surveillance system reported that influenza viruses circulated more frequently during periods of lower temperatures. Nevertheless, no modeling analysis has been presented to substantiate this claim.

The association between influenza transmission and climate has significant implications for public health, and an understanding of the period of influenza transmission could facilitate the anticipation of severe pathology and the vaccination of vulnerable groups. Secondly, it is unclear whether the disease circulates as a regional epidemic or is sustained by imported cases at the end of major human migrations, such as the Hajj or religious holidays.

The primary questions to be addressed in Sahelian areas like Niger are thus (i) what the timeline of transmission is and (ii) what climatic factors are driving this transmission.

## Methods

### Study setting and design

Niger is a large landlocked country in West Africa, covering an area of 1,267,000 km². It is situated between Algeria in the northwest, Libya in the northeast, Chad in the east, Nigeria in the south, Benin in the southwest, Burkina Faso in the west-southwest, and Mali in the west. The capital city of Niamey is situated along the Niger River in the southwestern region of the country, with coordinates of 13°30′49″ N latitude and 2°06′35″ E longitude. Niger experiments a Sahelian continental climate, comprising four principal seasons. The rainy season, which spans from June to September, is marked by elevated humidity and an average temperature of 33°C. The mid-hot season, occurring from October to mid-November, with a high relative humidity and an average temperature of 35°C. The cold season, from late November to February, which is characterized by cool nights with temperatures occasionally dropping below 10°C. The hot and extremely dry season, spanning from March to May, is marked by high temperatures and low humidity, with temperatures reaching 45°C in the shade during the day.

**Fig 1:**
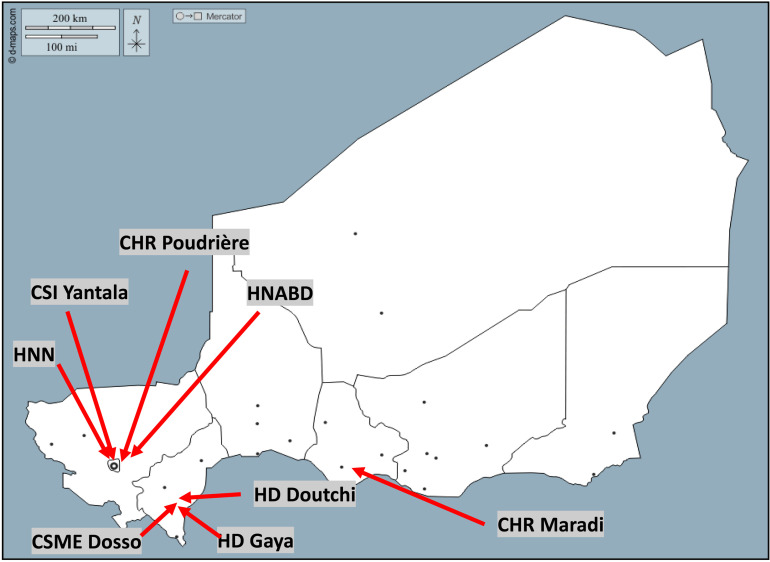
Localization of the sentinel sites in Niger.

The influenza sentinel surveillance network consisted of eight sentinel sites in total, spread across three distinct geographical regions, including the capital city of Niamey (see Fig 1). The present study encompasses the collection of influenza data from 2010 to 2021.

### Sample collection

From 2009 to 2017, nasopharyngeal swab samples were collected during the five working days at each sentinel site from ILI and SARI patients, in accordance with the WHO case definition of 2009. An ILI case was defined as an outpatient who presented with sudden-onset fever (≥38°C) and cough within the seven days prior to their presentation to a health facility (item i). A SARI case was defined as an inpatient who presented with fever (≥38°C) and cough within the seven days prior to hospital admission (item ii). Since 2018, cases of ILI and SARI have been enrolled based on the updated WHO case definition, which considers the history of fever, in addition to the onset of fever in SARI cases. The period of symptom onset was extended from seven to ten days for both ILI and SARI cases. (https://www.afro.who.int/publications/protocol-national-influenza-sentinel-surveillance).

Nasopharyngeal swab samples were collected on a daily basis by each sentinel site using Copan Universal Transport Medium (UTM-RT®). In addition, healthcare workers administered a standardized questionnaire to any suspected cases. The data collected encompass the demographic characteristics of the patient, their medical history, the date of enrollment, the date of symptom onset, their gender, age, and the clinical symptoms exhibited.

Prior to sampling, verbal informed consent was obtained from patients aged 18 years and above, or from the parents or legal guardians of minors. The sampling strategy was based on the collection of five samples per sentinel site per week, in order to align with the laboratory testing capacity. Nevertheless, this number may be augmented to ten in accordance with the period of elevated influenza transmission.

### Virological testing

Detections of Influenza A and B Virus were conducted at the National Influenza Reference Laboratory for influenza and other respiratory viruses through real-time reverse transcription polymerase chain reaction (qRT-PCR), utilizing reagents supplied by the Centers for Disease Control and Prevention (CDC) in Atlanta through the International Reagent Resources (IRR). Nucleic acid extraction was performed using the QIAamp mini kit (Qiagen, Germany) in accordance with the manufacturer’s instructions. Influenza A and B typing and subtyping were conducted using specific primers and probes in accordance with the CDC Atlanta protocol. All influenza A positive samples were subjected to subtype analysis for either A/H1N1pdm or A/H3N2. In contrast, influenza B positive samples underwent genotyping to detect the presence of the Yamagata and Victoria lineages. This methodology was employed in accordance with the procedures outlined by [13–14]. All influenza virological data are disseminated on a weekly basis via the WHO FluNet database.

### Meteorological data

Meteorological data were obtained from the Meteorology Directorate and include the following variables: minimum and maximum temperature (in degrees Celsius), wind speed (in meters per second), relative humidity (in percentage), and precipitation (in millimeters). The data, collected daily in Niamey, cover all eight sentinel sites. Given the relatively low daily detection rate of influenza viruses, the climatic data were gathered on a weekly basis, and the mean of each parameter was considered for analysis.

### Statistical approaches

The demographic characteristics of all tested patients were analyzed, along with the seasonal patterns of influenza virus circulation. This was conducted using Stata Version 16.1, produced by StataCorp in Texas, USA. The X² test was employed to ascertain any discrepancies in the proportions. A p-value of less than 0.05 was considered statistically significant. In this analysis, the week was identified as the elementary statistical unit, to which the climatic data, along with the total number of tested samples and influenza cases from that same week, were assigned.

All the association studies between climatic and influenza data were conducted using the R programming language, version 4.3.3 [15]. The heatmaps [16] are based on a hierarchical clustering with Ward’s aggregation criterion applied on Euclidean distance matrices, computed on centered and scaled raw climatic variables. Scaling was calculated over the twelve-year data set, ensuring that all the variables have the same weight in computing the distances between weeks. All the presented correlations are Spearman correlations.

In order to conduct generalized additive modelling (GAM), the gam function from the mgcv R package [17] was used. To facilitate comparison between years, the number of cases of influenza A and B detected each week was normalized by the total number of tests conducted in the same period. The GAM modeling analysis was limited to weeks with at least one case detected.

To identify the climatic conditions that most effectively explain the proportion of influenza cases, while accounting for cumulative effects, we computed a series of cumulative climatic parameters by calculating the cumulative sum (or mean for temperature and wind) of each parameter over a varying range of previous weeks (lag). The range for wind speed used, was from zero to 11 weeks, rain from zero to five weeks, humidity and minimum temperature from zero to eight weeks, maximum temperature from zero to nine weeks. This approach enabled us to study the cumulative impact of each climatic parameter over varying timeframes. Subsequently, a GAM model was fitted for each combination of these cumulative variables across all lag periods, resulting in a comprehensive collection of 22,328 (12 x 6 x 6 x 6 x 8) different model candidates. From this collection, the model that explained the greatest proportion of deviance was selected. This value reflects how much of the data’s variance is explained by the model, with a value of 100% indicating that the model fully explains the variance. To enable a comparative analysis of the explained deviances, a simplified GAM model, adjusted solely on the effect of the current week, has been firstly implemented. In this context, “explained deviance” refers to the proportion of the null deviance explained by the selected model. To compute the null deviance, only the offset is considered. For purposes of clarification, the deviance of a model is defined as -2*log(likelihood). The approximate significance of the smooth terms within the GAM model is evaluated using a Fisher test.

### Ethics approval

In 2009, the National Ethics Committee of Niger approved the national influenza surveillance program (reference No. 06/2009/CCNE of April 2009). In 2012, prior to the investigation of other respiratory pathogens, the Ministry of Health provided an extended approval. It should be noted that no nominal information of a personal nature was used during this study.

## Results

### Epidemiological data

All data were prospectively collected as part of the integrated influenza sentinel surveillance program through eight sentinel sites from 2010 to 2021 (Table 1, S1 Table). A total of 9,836 patients with influenza-like illness (ILI) and severe acute respiratory illness (SARI) were tested, of whom 982 (9.98%) were confirmed positive for either influenza virus A or B. Of these cases, 773 (78.71%) were children under five years. No statistically significant difference was observed between males and females. Of the total number of cases, 530 (53.97%) were classified as ILI cases. Of the influenza A/B positive cases, 631 (64.25%) were detected during the periods of low temperature, either the cold or the rainy seasons. However, the highest weekly positivity rate was observed during the cold season (15.3%) and the dry season (9.6%), which highlights the potential influence of other etiological factors during the rainy season (Table 1).

**Table 1.** Distribution of influenza cases by age group, sex, seasons and clinical manifestations.

Characteristics	INF A/B			p-value
	Negative	Positive	Total	
	(N = 8854)	(N = 982)	(N = 9836)	
**Age group (years)**				
< 1	3005 (30.55)	256 (2.6)	3261 (33.15)	<0.001
1-4	3851 (39.15)	517 (5.25)	4368 (44.4)	
5-14	828 (8.41)	108 (1.09)	936 (9.51)	
15-29	360 (3.66)	35 (0.35)	395 (4.01)	
30-44	314 (3.19)	28 (0.28)	342 (3.47)	
45-59	202 (2.05)	14 (0.14)	216 (2.19)	
>60	232 (2.35)	17 (0.17)	249 (2.53)	
Unknown	62 (0.63)	7 (0.07)	69 (0.7)	
**Sexe**				
Female	4025 (40.92)	462 (4.69)	4487 (45.61)	0.4
Male	4815 (48.95)	520 (5.28)	5335 (54.23)	
Unknown	14 (0.14)	0 (0)	14 (0.14)	
**Clinical manifestations**				
SARI	4793 (48.72)	441 (4.48)	5234 (53.21)	
ILI	3922 (39.87)	530 (5.38)	4452 (45.26)	<0.001
Unknown	139 (1.41)	11 (0.11)	150 (1.52)	
**Season**				
Dry saison	1966 (19.98)	209 (2.12)	2175 (22.11)	<0.001
Cold saison	2889 (29.37)	522 (5.3)	3411 (34.67)	
Rainy saison	2299 (23.37)	109 (1.1)	2408 (24.48)	
Hot saison	1691 (17.19)	141 (1.43)	1832 (18.62)	
Unknown	9 (0.09)	1 (0.01)	10 (0.1)	

INF A/ B: Influenza type A and B, SARI: Severe Acute Respiratory Infection, ILI: Influenza like illnes

### Seasonal patterns of the flu transmission in Sahel

**Fig 2:**
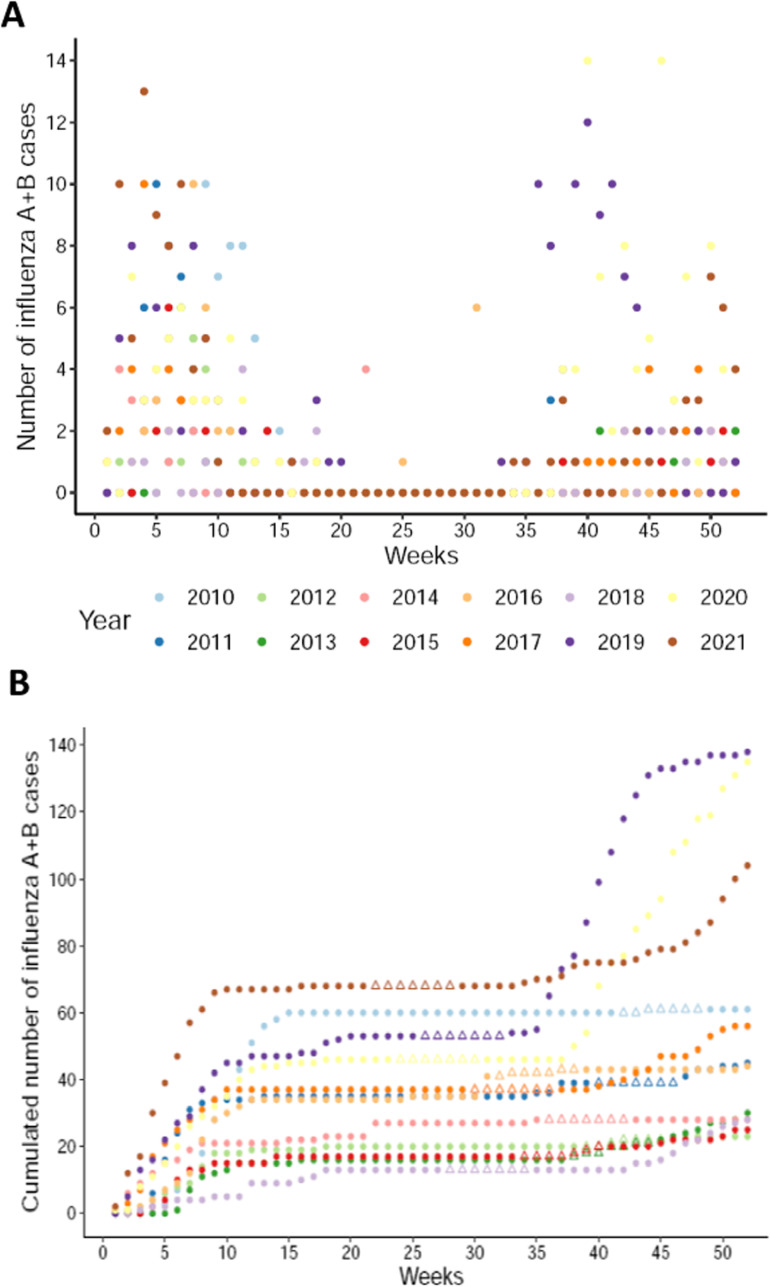
Number of influenza cases registered by week (2010-2021). (A) total weekly number of influenza A + B cases; (B) Cumulative number of influenza A + B cases along the year. All observation years are overlapped and identified by a distinct color. Triangles indicate the Hajj period each year, showing no correlation with the peaks in case numbers.

The climatic and microbiological data have been plotted annually for each year of collection (see Fig 2A and 2B). In the subsequent analysis, the “week” is thus the elementary statistical unit. Pics of influenza transmission demonstrate a high level of similarity from one year to another, as the occurrence of these events aligns with similar periods throughout the year (Fig 2A). Two periods which overlap towards the end of the calendar year (between weeks 40 and 15) account for the majority of cases. The year-to-year transmission of the virus started approximately in November, corresponding to the end of the rainy season. The number of cases increases rapidly during the dry winter season (December to February, Fig 2B). No evidence was identified that transmission shifts in response to holidays or cultural events (Fig 2B), such as the Muslim Hajj. Consequently, it may be inferred that a periodic component drives influenza cases. During the 2019-2020-2021 period, an increase in cases was observed, particularly during the period of the SARS-CoV-2 pandemic (Fig 2B).

### Association between influenza and climatic data

**Fig 3:**
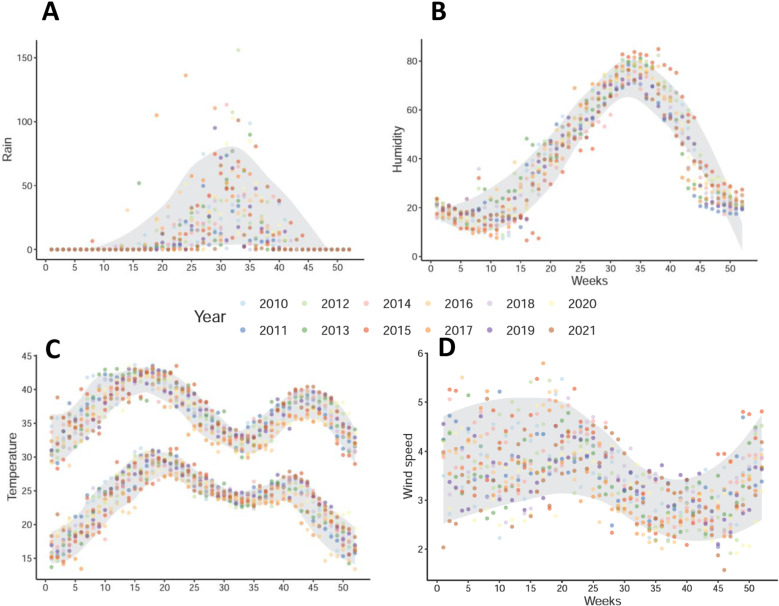
Weekly climatic data registered in Niamey (2010-2021). All observation years are overlapped and identified by a distinct color. (A) Average maximum temperature and minimum temperature of the week; (B) Average humidity registered during the week; (C) Average wind speed registered during the week; (D) Total rainfall registered during the week (Raw data are plotted, and a 95% probability interval is computed. A LOESS smoothing with a span parameter set at 0.2 is applied to derive the smoothed curves in grey.) Rainfall and wind are more variable from one year to another for the same week of the year.

To ensure a sufficient number of cases, weekly influenza data from the eight sites were combined, and only Niamey’s weekly meteorological data were used due to data availability (see Fig 3). A 95% probability interval based on empirical quantiles (2.5% - 97.5%) calculated over the twelve years has been included on the same plot as the raw data. During the winter months (December to February), the predominant wind is the Harmattan, a cold and arid wind originating from the north-east. Consistency between humidity and the onset of the rainy season was observed as well as a notable consistency in temperature patterns from one year to another. There was greater variability in wind speed and rainfall from year to year.

In a bivariate analysis, each week was treated as an independent unit, and a Spearman correlation was calculated between the weekly influenza case numbers and the corresponding climatic conditions. An inverse correlation was identified between influenza and rainfall (r = -0.36, p < 10e-3), humidity (r = -0.40, p < 10e-3) and minimum temperature (r = -0.34, p < 10e-3), while no correlation was observed with wind speed (r = -0.04, p = 0.34) or maximum temperature (r = -0.06, p = 0.17).

### Clustering of weeks according to global climatic conditions

**Fig 4:**
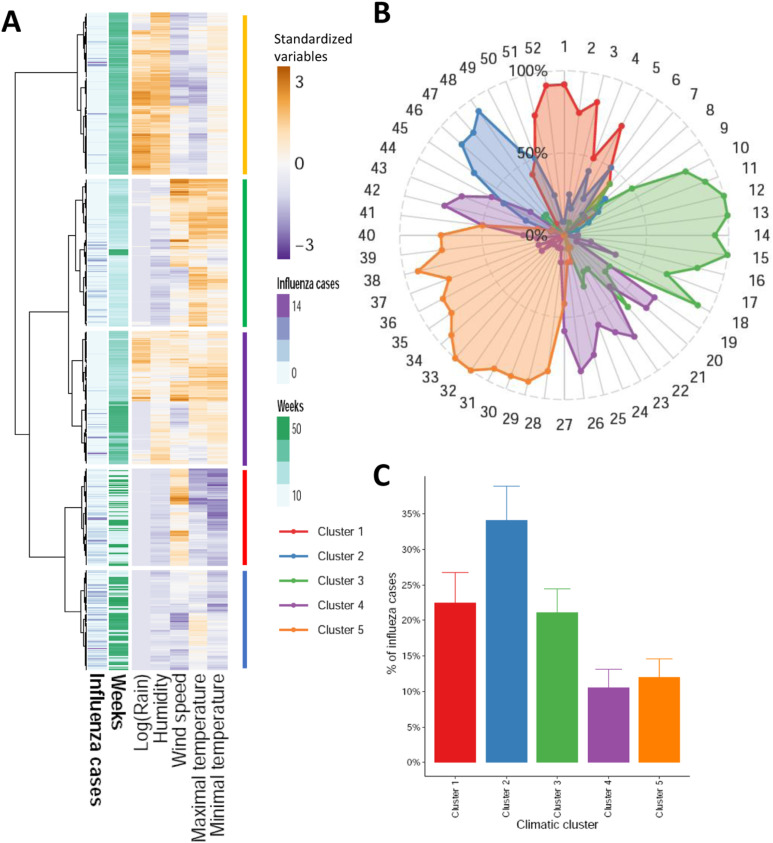
Identification of homogeneous climate periods based on weekly analysis. Each week is characterized by five parameters (Tmin, Tmax, humidity, wind speed, log of rainfall). A hierarchical clustering is performed to group weeks harboring the same profiles of climatic conditions. **(A)** Heatmap of clusters grouping the 624 weeks of the period of the study (52x12); five main clusters can be identified; the number of influenza cases by week and the week of the year are added to the heatmap as additional variable on the left. The colorscale indicates the climatic deviation of each week to the global mean of the 624 weeks: white color represents a climatic parameter close to the mean, dark orange corresponds to a high value of the climatic parameter and dark purple to a lower value. **(B)** Radarplot summarizing the contribution of each cluster over the year; for each week of the year the part of each cluster over the 12 years is reported in the plot. Overlaps of two clusters show that a week may belong to one cluster rather to another due to climatic variability of the week, especially during seasonal transitions (e.g., weeks 49-50). **(C)** Barplot reporting the proportions of total influenza cases by cluster, over the 12 years. It shows that most of influenza cases are in cluster 2 while cluster 4 captures less cases.

In order to study the potential association between global climatic parameters and the incidence of influenza cases, a multivariate approach was employed to identify distinct periods of homogeneous climatic conditions, irrespective of the specific time of year. A hierarchical clustering analysis was conducted on the 624 weeks included in the study. The distance between weeks was calculated based on the five related climatic parameters. The number of influenza cases registered for each week has been presented as supplementary annotations, but was not included in the clustering computation (Fig 4). The clustering analysis yielded five groups (clusters) of weeks, exhibiting a high level of homogeneity (as indicated by the branch heights in the clustering tree, see Fig 4A).

While the human concept of seasons is based on global climatic trends and defined by fixed beginning and end of a calendar period, it does not reflect the inherent variability of weekly weather patterns. In this context, the five groups identified via the aforementioned clustering algorithm may be interpretated as genuine seasonal clusters, reflecting the climatic conditions prevalent in Niger regardless of the calendar period.

**Fig 5:**
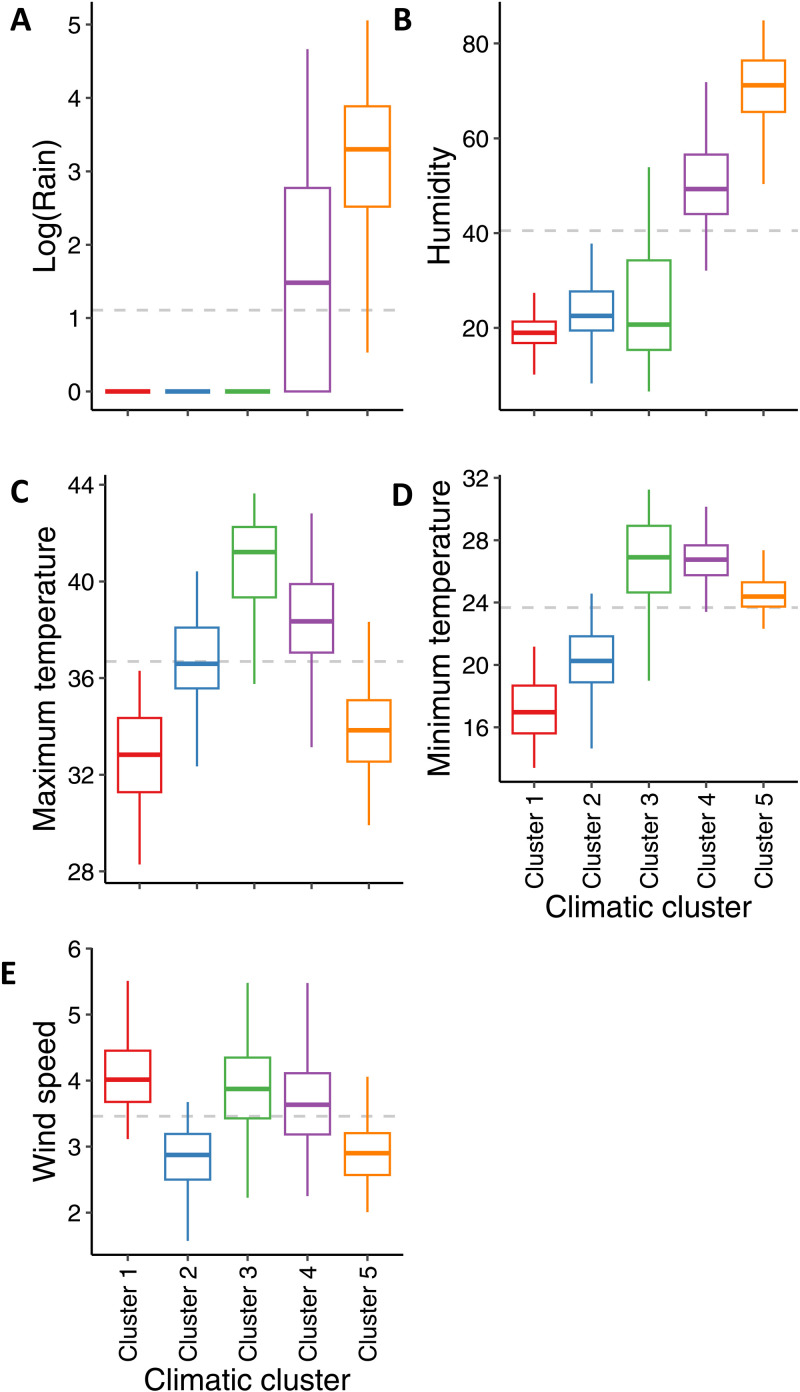
Description of climatic clusters by climatic parameters. Boxplots showing the distributions of **(A)** Log of rainfall, (B) humidity (C) maximum temperature (D) minimum temperature (E) wind speed, within each of the five climatic clusters. The grey dashed line represents the mean over the 624 weeks of the period of the study. Color of the box matches the cluster number. Weeks exceeding ±1.5 * (interquartile range) are spotted by individual dots.

To summarize the 12 years of the study, a radar plot (Fig 4B) represents the proportion in percentages of each seasonal cluster for every week of the year. The weeks where a cluster is predominant are not purely contiguous in the calendar as clusters aggregate weeks with similar climatic conditions over the year. Nevertheless, this annotation shows that each climatic profile occurs every year roughly at the same period of the year which confirms the strong periodic pattern of climatic data (Fig 3A-3B). The beginning and end of these clusters of climatic conditions can overlap depending on the year. Indeed, the first cluster occurs between the end and the beginning of the year with no rain, low temperatures with some weeks experimenting intense wind (Fig 3A and Fig 5). This period fits with the winter season with Harmattan wind characterized by low temperatures during the nights and dry wind with dust daytime. The second cluster is hotter but with low minimal temperature, dry without rain nor wind. It covers weeks of the end of the year, corresponds to the short dry-hot interseason (October to December) and has the most flu cases (Fig 3A and 3C). The third cluster is hot with hardly any rain except for few weeks, and variable wind. It occurs at the end of the dry season between week 11 and 18. It is the hottest and dryest period in Niger. The fourth cluster is hot, rainy, humid, with variable wind and with the fewest cases of flu (Fig 3A and 3C). It covers 2 periods of the year between cluster 3, 5 and 2. During this period the intertropical front rises up from the Guinea gulf to the Sahelian area bringing wet air and rain. It is the beginning of the rainy season which can variate from one year to another. The last cluster is colder, highly rainy with low wind and covers a large period of the year (13 weeks) but with few flu cases.

The percent of registered flu cases is also plotted for each cluster (Fig 3C) as barplots. Few flu cases are registered during the rainy period (clusters 4 and 5). A large part of the flu cases occurs during the winter cluster (cluster 1) and the dry and hot period (cluster 3). Some flu cases occur during the inter-season cluster (cluster 2). Some clusters are thus grouping most of the flu cases supporting a link between climatic conditions of a week and flu cases of the same week. Fig 5 summarizes the climatic parameters distribution of each cluster previously defined.

#### Association between influenza and cumulative climatic data.

Medical evidence suggested the potential for a cumulative effect of climatic conditions on the transmission of influenza. Low temperatures and dry winds have been shown to induce irritation of the respiratory system, thereby facilitating infection. Indeed, an analysis of the cumulative effect of climatic conditions could thus be relevant. The cumulative effect over a period between one up to twelve weeks prior to the reference week for influenza cases has been investigated with regard to the five climatic parameters. Given the temporal nature of the data and the suspected autocorrelation within and between time series, a Generalized Additive Model (GAM) was deemed appropriate for this analysis. A total of 624 weeks were analyzed, with average climatic parameters as explanatory variables and the number of recorded flu cases as the response variable. A collection of GAM models has been estimated to explain the proportion of flu cases, adjusted on both the number of the week and one of the 22,328 possible combinations of cumulative climatic conditions for the five parameters. From this model collection, the GAM model maximizing the percentage of explained deviance has been selected (see Materials and Methods for further details). This has led to the identification of the optimal combinations of parameters, as follows: mean maximum temperature for the last seven weeks (p = 0.055), mean minimum temperature for the last three weeks (p = 0.01), mean speed of wind for the last nine weeks (p = 0.048), sum of rain for the last three weeks (p = 0.002) and sum of humidity for the last four weeks (p < 0.001).

**Fig 6:**
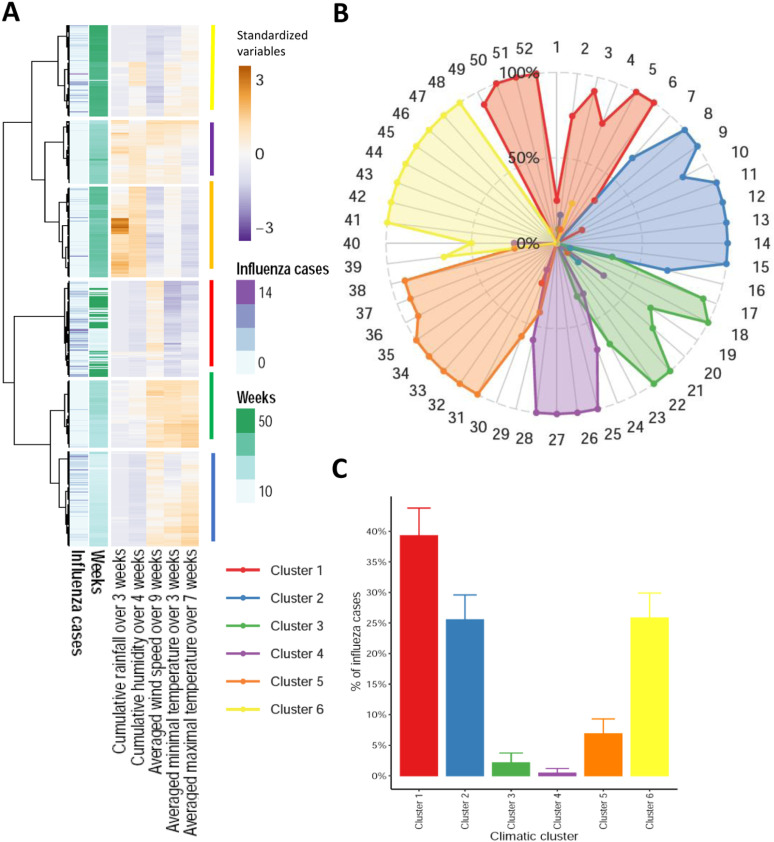
Identification of homogenous climate periods based on weekly analysis after GAM modeling of cumulated climatic effects. The number of influenza cases registered during each week is explained using a collection of GAM models fitted on all combinations of the five climatic parameters cumulated over 1 up to 12 weeks, i.e., cumulative rain, mean of maximum temperature, mean of minimum temperature, mean wind speed and mean of humidity. Model selection based on explained deviance identifies lags of 7 weeks, 3 weeks, 9 weeks, 4 weeks, 3 weeks for maximum temperature, minimum temperature, wind speed, humidity, rainfall respectively as the best combination of climatic parameters. A hierarchical classification as previously is applied to group weeks harboring the same profiles of cumulated climatic conditions. **(A)** Heatmap of clusters grouping the 624 weeks of the period of the study (52x12); six main clusters can be identified; the number of influenza cases by week and the week of the year are added to the heatmap as additional variable. The colorscale indicates the climatic deviation of each week to the global mean of the 624 weeks: white color represents a climatic parameter close to the mean, dark orange corresponds to a high value of the climatic parameter and dark purple to a lower value. Note that the global mean is computed on the cumulative climatic parameters over the corresponding lag. **(B)** Radarplot summarizing the contribution of each cluster over the year; for each week of the year the part of each cluster over the 12 years is reported in the plot. Note that the overlap between clusters is drastically reduced with clear chronological segregation of the weeks over the year. **(C)** Barplot reporting the proportions of total influenza cases by cluster, over the 12 years. It shows that most of influenza cases are in cluster 1 followed by clusters 2 and 6 while cluster 4 captures hardly any cases.

A hierarchical clustering analysis has been conducted on the 624 weeks of the study. For each week, the instantaneous climatic parameters recorded during that period were no longer taken into account, but the distances between weeks were calculated using the aforementioned cumulative versions of the climatic parameters (see Fig 5A). As illustrated in Fig 6, the number of influenza cases and the number of the week were plotted in the heatmap without contributing to the distance calculation and thus to the clustering.

Similarly to Fig 4, Fig 6 represents the climatic combinations that were most predictive of influenza cases according to the GAM modelling, and how they discriminate the influenza cases. This figure offers insight into the potential association between influenza and climatic data. The analysis identifies six updated clusters (Fig 6A), which correspond to periods of high or low influenza observations (Fig 6C). This allows the identification of periods with low influenza cases (cluster 4) or high influenza cases (clusters 1, 2, and 6). These clusters also exhibit a strong consistency with the seasons in Niger, as illustrated by the radar plot of the 52 weeks of the year (Fig 6B).

Fig 5, summarizes the distribution of climatic conditions of the six newly defined clusters. Using this approach, the seasons defined by these six clusters are much better characterized and do not overlap over the weeks. They therefore characterize well the periods of the year over the twelve years.

In summary, an analysis based solely on the periodic nature of the data (GAM model adjusted only on the number of the week) explains 11.1% of the variability in influenza cases (Fig. 2). Adding the climatic conditions of the current week increases the variance explained by the GAM model to 37.5% (Fig. 4), while identifying a relevant combination of lags specific to each climatic variable allows the explained variance to increase up to 77.3% (Fig. 5). Overall, this study strongly suggests that climatic conditions play a significant role in explaining influenza transmission in the Sahel.

## Discussion

Climate change is anticipated to modify the occurrence, prevalence, and distribution of infectious diseases due to its influence for example on temperature and precipitation levels [18–19]. It is essential that the National Control Programs and healthcare providers are aware of these alterations to enable them to anticipate adaptations to the strategy or timing of intervention [20]. However, there is evidence that the transmission of infectious diseases depends on the cumulative events that occurred several weeks earlier [20]. This is particularly the case for vector-borne diseases, but also for water- or wind-borne infections (as for meningitis [21–22]). A recent study has provided evidence supporting that higher temperatures and humidity levels were associated with more prevalent outbreaks of the Coronavirus Disease 2019 (COVID-19) pandemic [23]. For the flu transmission, this study confirms a clear impact of climate conditions in Niger. Six distinct seasonal periods are identified, with the most conducive conditions for the proliferation of influenza occurring in conjunction with dry cold and windy weather patterns. This study corroborates the hypothesis of a prior preliminary work conducted in 2010 in other continents [24–25]. However, the climatic conditions that occur during the weeks preceding the detection of clinical cases are of greater significance [26–27]. In our study, we fitted a statistical model that accounts for 77% of the variability of the occurrence of influenza cases, indicating that the epidemic can be anticipated weeks before clinical detection in healthcare structures.

A variety of techniques can be employed to examine the relationship between case reports and meteorological conditions. Time series analysis models offer a particularly robust foundation for such investigations. However, these models can become difficult to manage when multiple points of survey distributed across a country are analyzed simultaneously. In this study, a more user-friendly methodology was implemented, enabling efficient analysis across a vast region and over multiple years. Each week was treated as a discrete statistical entity. Multidimensional statistics allow the identification of homogeneous groups of geographical points of survey and/or the reconstruction of novel homogeneous seasons spanning multiple years.

However, factors can affect the relevance of the collected data and the connection between climatic conditions and diseases. Samples collected in reference centers in relation to severe diseases are not representative of the transmission of a disease, which can manifest with a low level of symptoms and not be associated with clinical consultation, even more with hospitalization [28]. Similarly, the intensity of sampling in a healthcare structure may be contingent upon the willingness of healthcare professionals to participate. Again, this will modulate the collection of positive samples in relation to this willingness and not with climate.

Another challenge is the collection of climatic data themselves, typically collected by national stations, but with low density across the country. This issue has been highlighted by a study conducted in Brazil, which proposed an alternative system of climate data collection based on satellite-generated climatological data to support agricultural research and development [29]. Local conditions may differ significantly from the standard conditions collected in the stations, which may result in an incomplete representation of the local ecology. Consequently, the impact of local weather conditions may be inadequately integrated into predictive models, particularly in cases where wind and dust affect the upper airways or rainfall influences mosquito proliferation.

Collection of data is thus always biased, and interpretation of the results must always go back to the sampling strategy to give accurate interpretation. However, in addition to climatic conditions, the movement of people is another significant factor contributing to the spread of infectious diseases. The primary human migrations in West Africa are associated with religious pilgrimages, festivals (Hadj) and herd transhumance [30]. Such movements may also exhibit seasonal regularity, which may coincide with climatic variations. However, in the Muslim area (where the calendar follows the lunar cycles), analysis will be more straightforward, as the main celebrations will have a 12-day lag from one year to another with the solar calendar.

At last, several etiologies can lead the same clinical syndrome, inducing confusion in analysis. Indeed, during this follow-up over the last twelve-year, influenza-like illness (ILI) cases accounted for only 10% of all clinical cases of respiratory illness. This indicates that further biological investigations are required to identify the remaining causes, which are likely to include bacteria, as supported by previous studies on the etiologies of severe acute respiratory illness (SARI) in Niger. This will be crucial for optimizing the medical response to these syndromes and for detecting new emerging pathogens. The incidence of influenza remained consistent from one year to the next, with a notable increase observed from 2019 to 2021. This can be attributed primarily to the implementation of integrated surveillance for the novel coronavirus disease (COVID-19) since 2020.

To conclude, this study on influenza transmission, can be limited in three significant ways. Firstly, the level of sentinel surveillance activity was not consistent throughout the study period, particularly from 2010 onwards. As a result, the data from 2018 are more representative, which can be attributed, at least in part, to funding from the WHO and the CDC Atlanta [31]. Secondly, the low weekly detection rate of influenza made it challenging to establish a precise correlation with the various climatic factors. Indeed, the annual positivity rate was less than 10%, and the average number of confirmed cases per week was relatively low, even reaching zero after the cold season. Finally, it should be noted that the climate data were predominantly sourced from a single station, which may not fully align with the data recorded at in sentinel sites. However, this survey has confirmed the seasonal transmission of influenza, with a clear impact of climate conditions. Nevertheless, a degree of variability persists, which can be influenced by human-related factors, including travel, migration and social gatherings. It demonstrates also that clustering and GAM models represent an effective and feasible method for examining the influence of climatic conditions on the transmission of influenza and other diseases. However, in the case of an epidemic with high transmissibility, the impact of human factors modelling human-to-human contact could be more significant. This could be achieved through the implementation of stochastic models for modelling the dynamic of the infection.

## Supporting information

S1 Table File containing data collected in the sentinel sites over the period of study (XLSX)
